# Structural Rearrangements of Pigeon Cryptochrome 4 Undergoing a Complete Redox Cycle

**DOI:** 10.1021/acs.jpcb.4c00424

**Published:** 2024-04-03

**Authors:** Fabian Schuhmann, Jessica L. Ramsay, Daniel R. Kattnig, Ilia A. Solov’yov

**Affiliations:** † Institute of Physics, Carl von Ossietzky Universität Oldenburg, Carl-von-Ossietzky Str. 9-11, Oldenburg 26129, Germany; ‡ Niels Bohr International Academy, Niels Bohr Institute, University of Copenhagen, Blegdamsvej 17, Copenhagen 2100, Denmark; § Living Systems Institute and Department of Physics, University of Exeter, Stocker Rd., Exeter EX4 4QD, U.K.; ∥ Research Centre for Neurosensory Science, Carl von Ossietzky Universität Oldenburg, Carl-von-Ossietzky-Str. 9-11, Oldenburg 26129, Germany; ⊥ Center for Nanoscale Dynamics (CENAD), Carl von Ossietzky Universität Oldenburg, Ammerländer Heerstr. 114-118, Oldenburg 26129, Germany

## Abstract

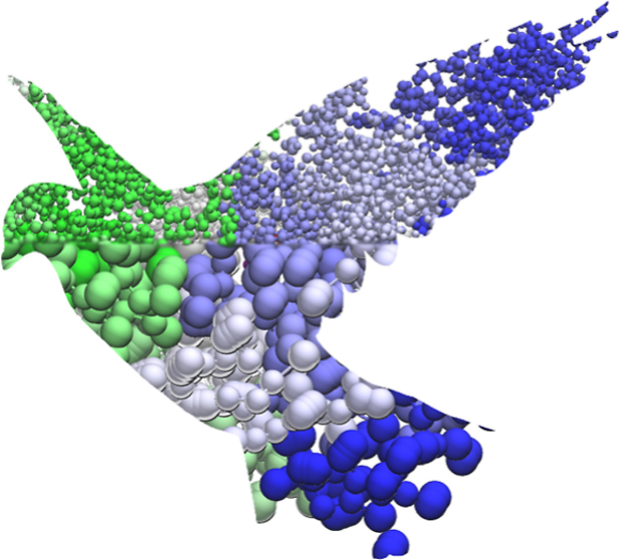

Cryptochrome is currently the major contender of a protein to underpin magnetoreception, the ability to sense the Earth’s magnetic field. Among various types of cryptochromes, cryptochrome 4 has been identified as the likely magnetoreceptor in migratory birds. All-atom molecular dynamics (MD) studies have offered first insights into the structural dynamics of cryptochrome but are limited to a short time scale due to large computational demands. Here, we employ coarse-grained MD simulations to investigate the emergence of long-lived states and conformational changes in pigeon cryptochrome 4. Our coarse-grained simulations complete the picture by permitting observation on a significantly longer time scale. We observe conformational transitions in the phosphate-binding loop of pigeon cryptochrome 4 upon activation and identify prominent motions in residues 440–460, suggesting a possible role as a signaling state of the protein or as a gated interaction site for forming protein complexes that might facilitate downstream processes. The findings highlight the importance of considering longer time scales in studying cryptochrome dynamics and magnetoreception. Coarse-grained MD simulations offer a valuable tool to unravel the complex behavior of cryptochrome proteins and shed new light on the mechanisms underlying their role in magnetoreception. Further exploration of these conformational changes and their functional implications may contribute to a deeper understanding of the molecular mechanisms of magnetoreception in birds.

## Introduction

Every year, birds migrate across the globe with remarkable precision, linking their breeding and wintering grounds.^1^ Decades of behavioral research have shown that migratory birds are sensitive to the Earth’s magnetic field, which has led to a surge in the search of the underlying sensory mechanism, both by theoretical and experimental approaches.^2−6^

The current understanding of the magnetic compass sense favors a model in which the birds’ astonishing feat of navigation is thought to be accomplished through the blue-light excitation of a flavin adenine dinucleotide (FAD) cofactor, noncovalently bound in cryptochrome 4 proteins in the animals’ retinae. This photoexcitation triggers the radical pair formation, where initially a radical pair is formed between the FAD and one closely located tryptophan in the protein. Subsequently, a cascade of electron transfers follows, resulting in a radical pair between FAD and a solvent-exposed tryptophan residue. However, the exact process of how the protein’s signals are interpreted by the brain remains unclear.^5^

Modeling the cryptochrome protein structure and employing molecular dynamics (MD) simulations allow the identification and quantification of conformational changes that might occur upon activation or reoxidation of the protein. Such motions can then be interpreted and used to suggest or explain functions of the protein.^7^

The initial radical pair formed within the cryptochrome 4 protein has a lifetime on the order of 1 μs. It is this radical pair that is thought to be influenced by the magnetic field, evidenced in the magnetic field sensitivity of the yield of chemically different product states.^8−13^ The ratio between these product states putatively allows the bird to sense the Earth’s magnetic field and navigate accordingly.^5,11,14^ Radical pair formation induces a structural change in the protein, specifically in a versatile region, the so-called phosphate-binding loop, as shown in an earlier study^7^ through all-atom MD simulations. It was suggested that upon activation, the phosphate-binding loop of pigeon cryptochrome 4 swings open like a gate, allowing solvent and potential reactants access to the FAD inside the protein. The solvent accessible surface area (SASA) of FAD was shown to double during the course of the activation.

The reoxidation, i.e*.*, the back-reaction that returns cryptochrome to its preactivation state, has received less interest in the past, even though it is potentially closely related to downstream signaling. This back-reaction is known to occur on the time scale of hundreds of microseconds,^5,15^ which cannot be easily studied through all-atom MD simulations, calling for alternative simulation approaches to be employed.

We employ a coarse-grained approach to study the conformational changes occurring in cryptochrome 4 on a longer time scale. The FAD and tryptophan are simulated in the forms relevant to their (re)oxidation cycle to study the effects of each redox state on the overall structure of the cryptochrome protein structure. Through this approach, conformational changes can be probed on the longer time scale required for the oxidation process. It was found that the phosphate-binding loop indeed performed a closing motion restricting the access of solvent to the FAD again. An additional region of interest was found, in which specific charged amino acids shift their position on the cryptochrome protein surface. A video of the full simulation cycle can be found in the Supporting Information.

### Activation Pathway

**Figure 1:**
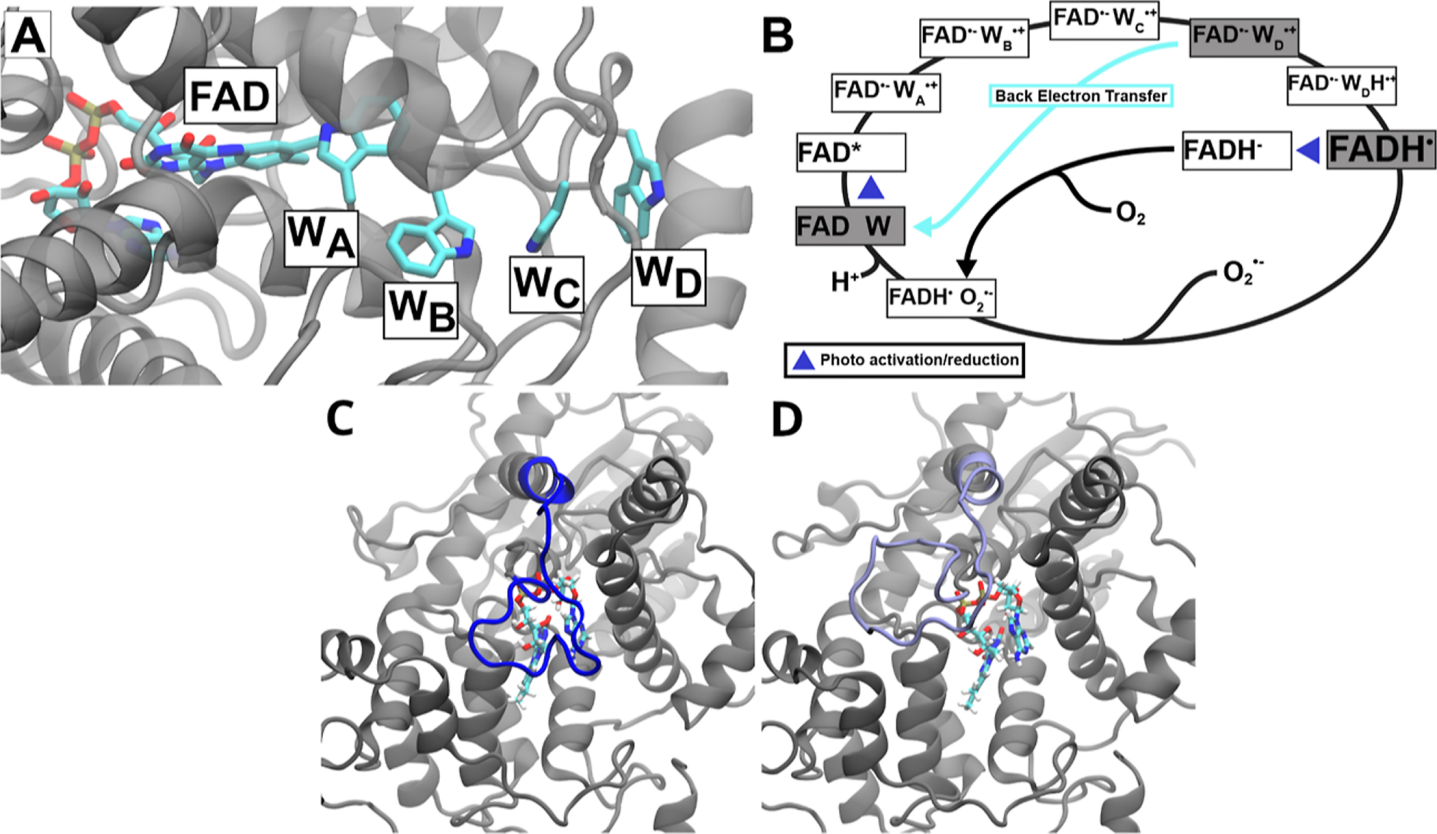
(A) FAD and the tryptophan tetrad are shown in their protein structure environment. Upon light-activation of the FAD, W_A_ donates an electron to the FAD, W_B_ gives an electron to W_A_ and in turn accepts one from W_C_. Finally, W_D_ donates an electron to W_C_. (B) Radical pair formation pathway and the reoxidation is shown, forming a cycle including the different radical pairs between the FAD, the respective tryptophan, and the FADH^•^, which is a potential signaling state. Panel (B) illustrates the redox cycle of the system. The three gray boxes highlight the specific redox states of cryptochrome that were simulated. (C) Closed phosphate-binding loop configuration is shown as seen in the all-atom simulation. (D) Open phosphate-binding loop configuration is shown.

Cryptochrome 4 is activated through blue light, which excites the FAD cofactor located inside the protein. Upon photoexcitation, an electron in the FAD is transferred to a higher energy level, which produces an electron vacancy and turns the FAD into an electron acceptor. A nearby tryptophan residue (W_A_) serves as the electron donor, replenishing the hole. This forms a spin-correlated radical pair consisting of FAD^•–^ and W_A_H^•+^, henceforth denoted as radical pair A (RPA). This radical pair is the first step of a cascade involving a total of four tryptophan residues (subsequent ones being denoted W_B_, W_C_, and W_D_), which pass electrons to their neighbors until finally the spin-correlated radical pair comprising FAD^•–^ and W_D_H^•+^ is formed, typically denoted as radical pair D (RPD).^5,7^ The four tryptophan residues and the FAD are shown in their environment within cryptochrome 4 in Figure 1A.

In its semireduced form, FAD^•–^ gets eventually protonated by an adjacent acid (probably H_2_O).^16^ FADH^•^ is suspected to be implicated with the signaling state of the cryptochrome 4 protein.^5^ The competition between protonation and back-electron transfer is central to the magnetosensitivity of the activation.^5^ The formation of FADH^•^ is also the beginning of a reoxidation process which reestablishes the dark state (DS) and primes the protein to start the radical pair formation process anew.^4,15,17^ Another redox state associated with the FAD is the fully reduced FADH^–^,^18−20^ which is not considered in this study; it returns to the FADH^•^ state via the means of oxygen^20,21^ to finally return to the fully oxidized form. While the RPD state has an expected lifetime of up to 1 μs,^8^ the reoxidation pathway of the FAD acts on a scale of more than 100 μs.^5,15^ On the other end, W_D_H^•+^ is deprotonated swiftly and subsequently reduced to reform W_D_H.^4^ The schematic radical pair reduction–reoxidation cycle is shown in Figure 1B. It has been studied experimentally in cryptochromes from different species.^5,15,20,22,23^

## Methods

### Molecular Dynamics

**Figure 2:**
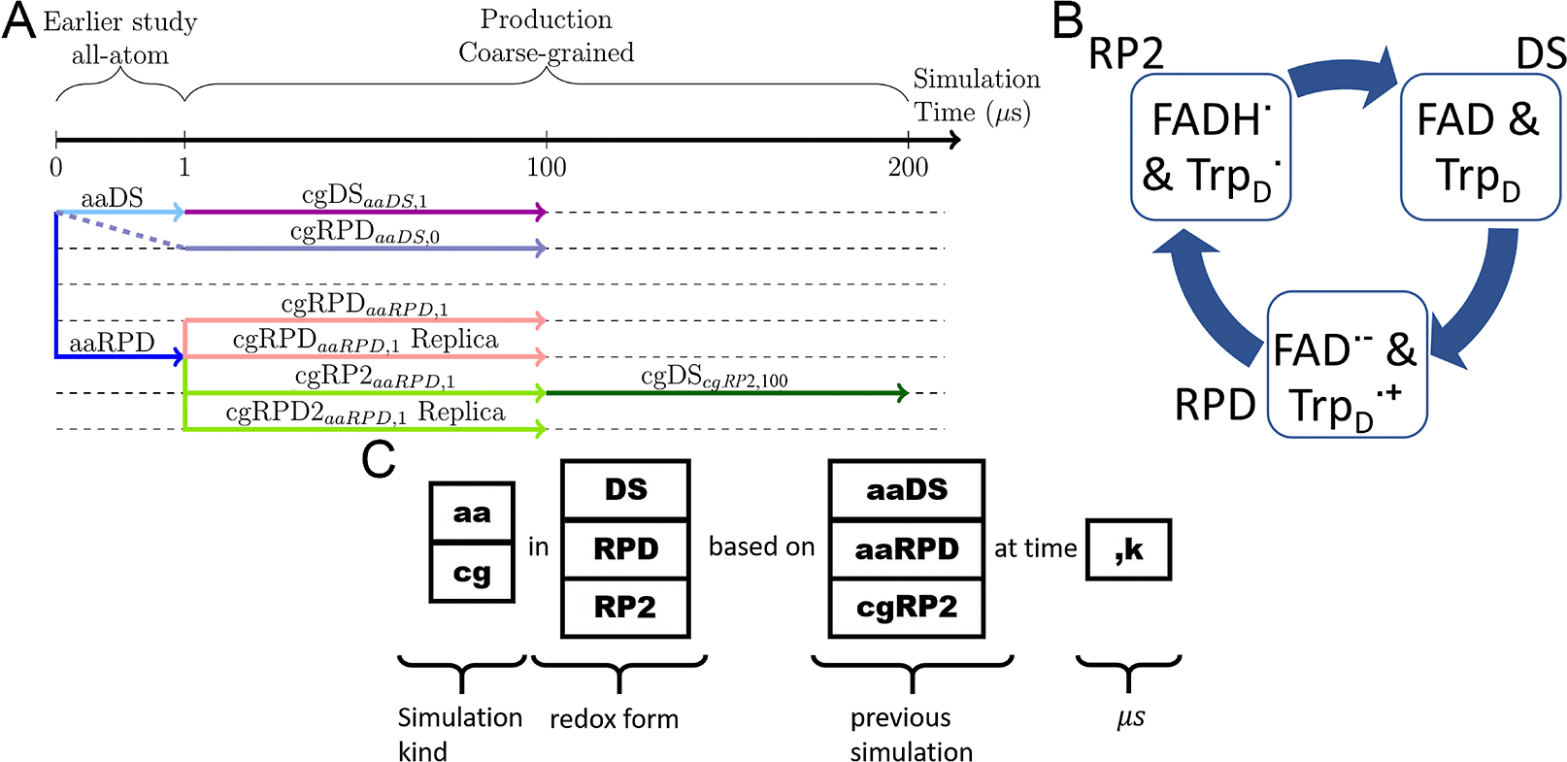
(A) Timeline for the conducted simulations of ClCry4 in the different states. The simulations labeled aaDS and aaRPD are all-atom simulations taken from an earlier study.^7^ From the first snapshot of the original DS simulation, a coarse-grained RPD state was initiated (cgRPD_*aaDS*,0_). The DS and RPD all-atom simulation were coarse-grained and prolonged (cgDS_*aaDS*,1_ and cgRPD_*aaRPD*,1_). From the last frame of the all-atom RPD simulation, a coarse-grained simulation was started, in which (FAD^•–^, W_D_H^•+^) were transformed to (FADH^•^, W_D_^•^), denoted as cgRP2_*aaRPD*,1_. After 100 μs, the state was changed once more to (FAD, W_D_), denoted by cgDS_*cgRP*2,100_, which coincides with the original DS. (B) Names for the different redox forms are introduced. The starting system is considered to be a DS, comprising FAD and W_D_. The correlated radical pair resulting from activation, FAD^•–^ and W_D_H^•+^, is denoted as RPD. The intermediate step in the reoxidation process involving FADH^•^ and W_D_H^•^, and later FADH and W_D_H, is denoted as RP2. (C) Label taxonomy for the different conducted simulations is introduced. For instance, cgRPD_*aaRPD*,1_ is the coarse-grained simulation of ClCry4 in the RPD form based on the structure from the all-atom RPD simulation after 1 μs.

ClCry4^19^ has been simulated using all-atom MD in an earlier study.^7^ The phosphate-binding loop was shown to be versatile and assumed either a *closed* state (Figure 1C), shielding the FAD binding pocket, as exhibited in an inactivated DS, or in an *open* state (Figure 1D), as seen in the all-atom RPD simulation. In order to explore a wide range of conformational behavior of the protein, multiple coarse-grained simulations were generated, initializing the system in *open* and *closed* starting configurations, reflecting different stages of the activation cycle. The conducted simulation plan is schematically shown in Figure 2A. The simulations are named based on the simulated redox form of the FAD and W_D_ (visualized in Figure 2B) and the corresponding initial configurations of the simulated system. The full naming taxonomy is explained in Figure 2C.

The ClCry4 protein was coarse grained employing the Vermouth Python package and its Martinize2 program,^24−27^ with the Martini22p force field. Parameters and Martini bead types to describe the FAD and FADH^•^ were taken from an earlier study.^28^ The different redox forms of the FAD cofactor and tryptophan residues were obtained by manually altering the FLA2 bead in FAD and the SC2 bead in tryptophan to reflect the different charges associated with different redox states. Martini^29^ was employed to generate the topology for virtual sites, which were needed for a stable structure. The model also assists in preserving the secondary structure of the protein, which is more difficult to quantify due to the missing backbone atom information. The virtual sites introduce nonbonded Lennard-Jones interactions with a potential energy well depth of ε = 9.414 kJ/mol. The overlap map and the repulsive contact and structural unit (rCSU) map were generated by the rCSU online platform^30^ for the RPD and DS starting configuration individually.

The original all-atom MD simulation conducted earlier^7^ was used to initiate multiple coarse-grained simulations describing different forms of the protein in its activation cycle. A full simulation plan, the defined redox states, and the labeling taxonomy for the different simulations are shown in Figure 2.

The all-atom structure with the *closed* phosphate-binding loop configuration (obtained from the all-atom DS simulation) was used to construct the coarse-grained DS model (cgDS_*aaDS*,1_). An RPD form (cgRPD_*aaDS*,0_) was also constructed from an all-atom configuration with the *closed* phosphate binding loop to examine whether the loop would move as previously seen in the all-atom simulation.

The all-atom structure with the *open* phosphate-binding loop configuration, as seen in the all-atom RPD simulation, was used to define the initial structure for the reoxidation pathway. Two coarse-grained replica simulations of ClCry4 were carried out in the form containing FADH^•^ and W_D_, denoted as cgRP2_*aaRPD*,1_ and Replica in Figure 2, and were used to model the reoxidation in ClCry4. The coarse-grained model does not distinguish between W_D_^•^ and W_D_H, i.e., the actual redox state of the W_D_-residue is immaterial. One of the two replica simulations was used to alter the redox state of the FAD cofactor once more to represent the original DS redox state of the protein (fully oxidized). This cgDS_*cgRP*2,100_ simulation was studied for another 100 μs. From the all-atom configuration with the *open* phosphate-binding loop configuration, two cgRPD replica simulations were executed (FAD^•–^, W_D_H^•+^), denoted as cgRPD_*aaRPD*,1_. The complete naming scheme for the different forms is given by the diagram shown in Figure 2B,C.

The coarse-grained structures were minimized in vacuum using GROMACS^32−39^ and subsequently solvated using the Martini water.^27^ Ten percent of the water beads were replaced with antifreeze water, which uses a slightly larger bead size and an adjusted Lennard-Jones potential. The addition of antifreeze water prevents clustering and freezing of the coarse-grained water beads, which otherwise has been shown to occur at temperatures from 280 to 300 K for Martini coarse-grained simulations.^27^ One water bead accounts for four water molecules. Six hundred and fifty three water beads were replaced with Na^+^ and Cl^–^ ion beads to neutralize the system and then achieve a salt concentration of 0.2 mol/L. An ion bead describes three water molecules and one ion. The solvated structures were minimized once more prior to the initiating of the MD simulations.

The simulations were carried out using GROMACS.^32−39^ The structures were equilibrated in an *NPT* (constant number of particles, pressure, and temperature) ensemble with positional restraints on the backbone beads. The temperature was set to 310 K. The equilibration simulation was conducted with a time step of 10 fs and a LINCS order of 8.^40^ After 300 ns of equilibration simulation, the ensemble was switched to *NVT* (constant number of particles, volume, and temperature), and the restraints were turned off. The equilibration simulation was then continued for an additional 500 ns.

The production simulations used for analyses employed the *NVT* ensemble until the desired simulation length of 100 μs was reached. The simulation time step was set to 20 fs. Finally, in the last step, all trajectories were unwrapped in regards to their periodic boundary condition using the GROMACS tool “trjconv”.^34^

### Stability

The software VMD^31^ was used to conduct a root-mean-square deviation (rmsd) analysis to assess the stability of the protein simulations. All trajectories were aligned with the first snapshot of the cgDS_*aaDS*,1_ simulation. The rmsd was then computed for the whole protein backbone and separately for the phosphate-binding loop only without any further alignment. The earlier study^7^ showed the phosphate-binding loop to be versatile, so the rmsd was employed to check whether this attribute is still consistently reflected in the coarse-grained situation.

### Trajectory Comparison

To compare the conformational changes in the ClCry4 protein and to pinpoint some of its versatile regions, Kullback–Leibler divergence (KLD) between the distributions of positions of each amino acid residue was employed. KLD is a measure that tends to suppress noise, which makes it suitable for the comparison of equilibrium MD simulations. KLD is calculated for two trajectories *Q* and *P* describing protein’s time evolutions for each amino acid residue *i* as
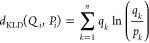
1where *Q*_*i*_ and *P*_*i*_ denote the distribution of locations of residue *i* over the trajectories *Q* and *P*, respectively; i.e., *q*_*k*_ is the *k*th value in the discretized distribution *Q* (analogously for *P*). *n* is the number of simulation snapshots used for comparison. A symmetrized measure is desirable to compare two trajectories and can be introduced as
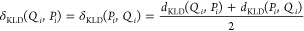
2

The positions of residues are represented by their respective backbone beads’ locations. The KLD measure is available through the software package SiMBols,^41^ which was utilized for the calculation. SiMBols also includes preprocessing steps for pairwise alignment of to-be-compared trajectory pairs. The alignment was done iteratively, as successfully employed in earlier studies,^7,42^ and the cgDS_*aaDS*,1_ simulation was used as the reference. The mathematical details of KLD are rigorously explained in the SiMBols documentation.^41^

Relying on the same alignment, KLD was calculated for windows of the simulations, allowing a time-dependent analysis of the comparison. Specifically, the similarity measure was computed for a moving window of a maximum of 50 simulation snapshots each. For instance, in the 49th calculation window, simulation snapshots 1–49 were considered. The next window considered snapshots 1–50 and then 2–51, and so on. Finally, the window size decreases again when the end of the simulation trajectories was reached.

### Solvent Accessible Surface Area

In order to further characterize the effects seen in the rmsd and comparison analyses, the time evolution of the SASA of the FAD inside the protein structure was calculated using the GROMACS sasa tool^36,43^ with a probe size of 0.26 nm corresponding to a regular Martini coarse-grained bead.

### Distance Analyses

**Figure 3:**
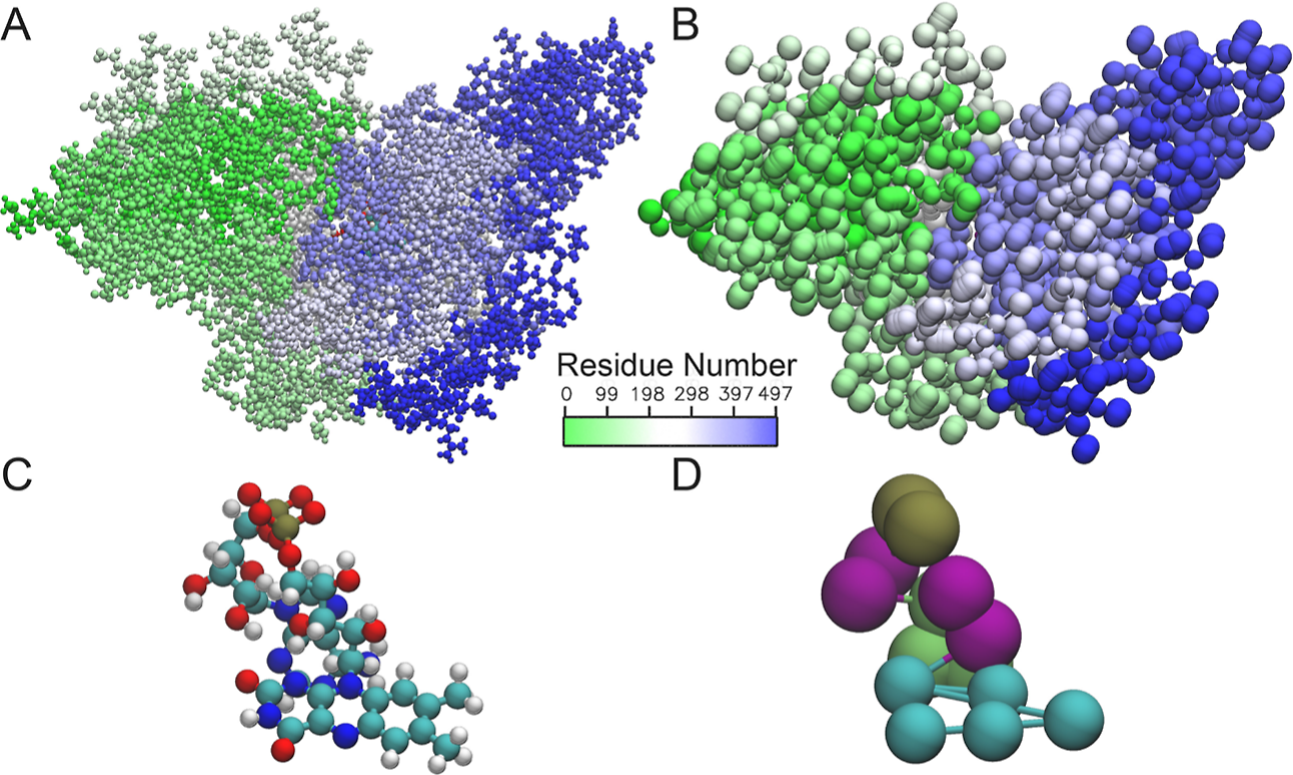
(A) All-atom structure of ClCry4 is shown. The structure has a total of 8090 atoms including hydrogens. The residues are colored according to their residue number from the beginning of the amino acid sequence (green) to the end (blue). (B) Coarse-grained version of the ClCry4 is shown. The structure contains a total of 1472 beads. The residues are colored analogously to the all-atom structure. (C) FAD is shown in its all-atom representation colored according to the chemical elements. (D) Coarse-grained FAD is shown colored according to the bead type, as obtained earlier.^28^ The image has been rendered using VMD.^31^

The distances between the FAD cofactor and the pertinent tryptophan residues are central to the magnetic field sensitivity of the radical pair^9^ and are in part experimentally accessible.^5,19^ We have extracted the distances from the flavin to the four individual tryptophan residues involved in the electron transfer tetrad, see Figure 1A. These distances are of particular importance as they directly modulate the electron transfer rates vital for the functionality of the protein.^5^ The distances obtained from the coarse-grained simulation can be compared to the distances calculated from all-atom simulations. Hanić *et al.*(44) have reported the corresponding distances measured for all-atom MD simulations, employing the center of mass of the flavin and the tryptophan residue as the anchoring points. Hence, the distances for the coarse-grained simulations were calculated similarly using the center of mass of the flavin, consisting of beads FLA1, FLA2, FLA3, FLA4, and FLA5 and the center of mass of the tryptophan residues (Figure 3).

## Results and Discussion

**Figure 4:**
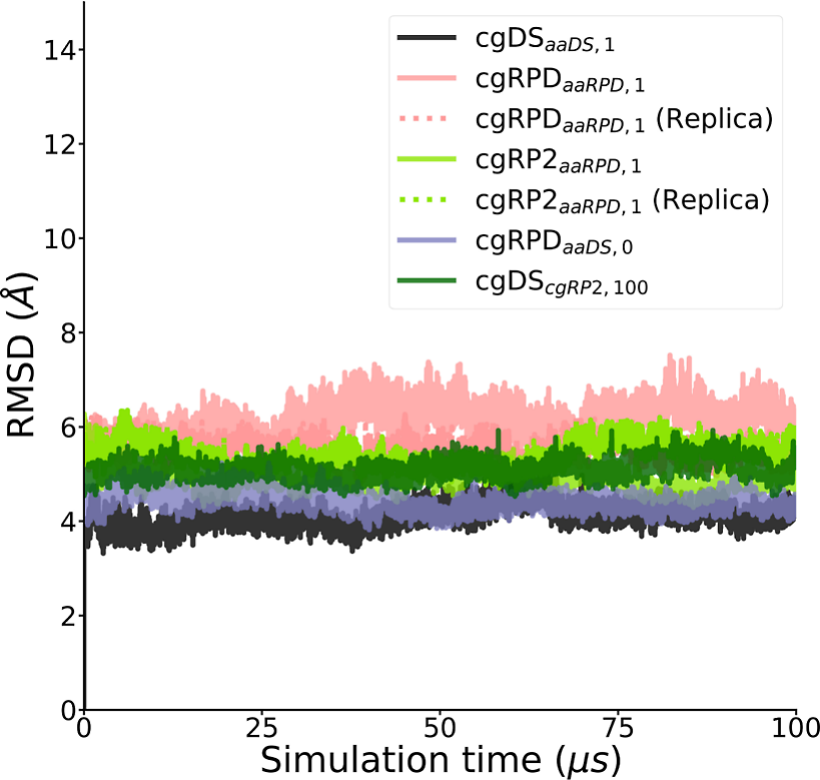
Time evolution of the rmsd computed for all simulation trajectories referenced and aligned to the cgDS_*aaDS*,1_ simulation as described in the text. The rmsd for the whole backbone of the protein is shown. The values remain at the same level throughout the simulation indicating a stable structure.

The rmsd showed general stability of the overall protein structure in all simulations. The rmsd values recorded for the cgDS_*aaDS*,1_ simulation are in good agreement with the all-atom counterpart of around 4 Å, as found in the earlier study.^7^ All of the conducted simulations did not show an unproportional increase in rmsd, which indicates stable simulations without a significant unfolding of the protein. The rmsd for the whole protein structure is visualized in Figure 4. Furthermore, the stability of the α-helical secondary structure motif was analyzed via rmsd time evolution. As the helices tend to be stable, an increase in rmsd would indicate an overall increased flexibility and loss of secondary-structure in the coarse-grained simulation. The average rmsd of the α-helical regions as classified in the all-atom structure is 4.48 Å compared to the value of 4.17 Å computed for the whole structure. The minor difference between the two averages indicates stable α-helical motifs and the absence of a significant decay of secondary structure in the coarse-grained simulation. The averages for the cgRP2_*aaRPD*,1_ and cgDS_*cgRP*2,100_ show an rmsd average for the α-helical regions of 5.17 and 3.24 Å, respectively, again in line with the value obtained for the whole-protein rmsd. The stability of the protein structure allows a further analysis quantifying conformational changes in the MD trajectories.

**Figure 5:**
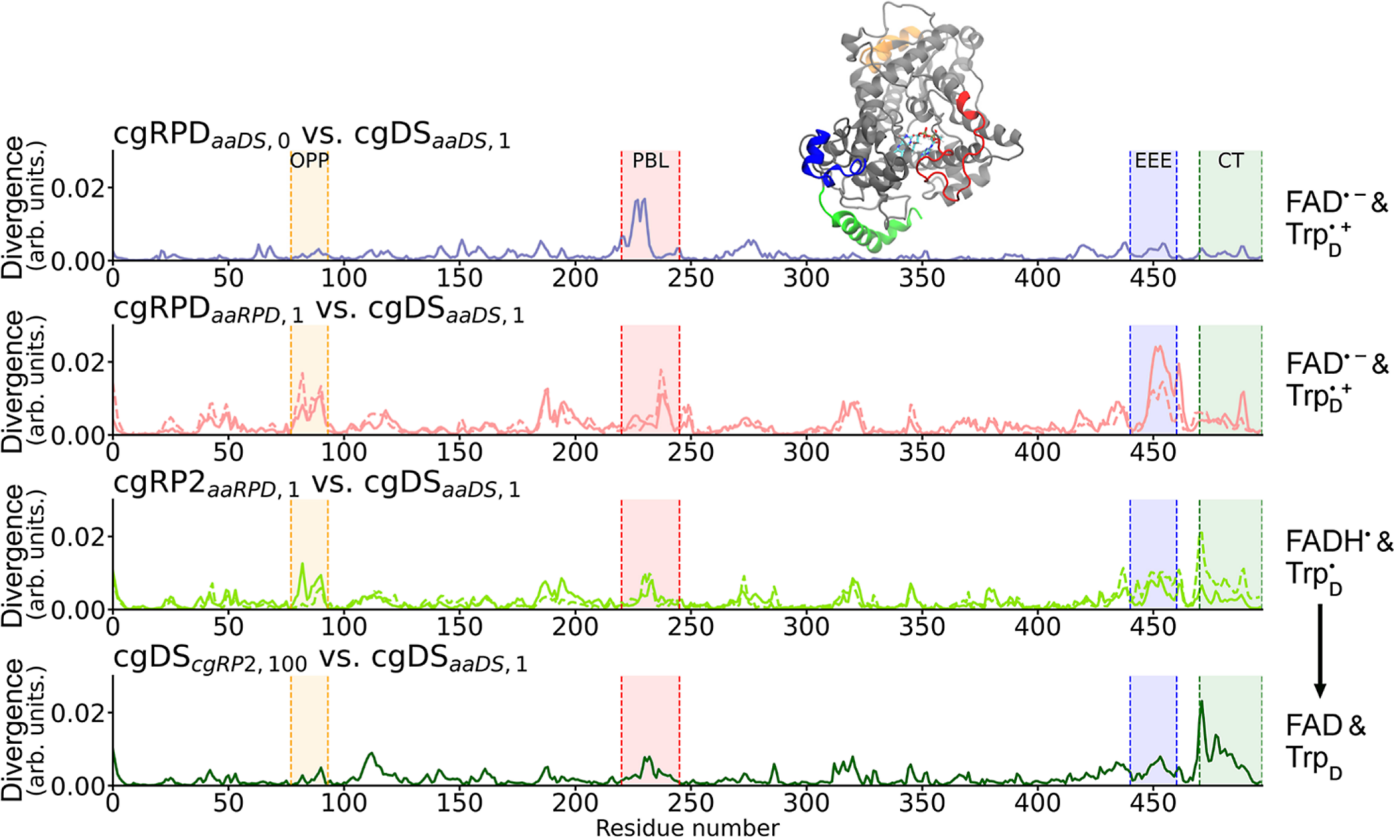
KLD comparison of the coarse-grained trajectories. Each simulation is compared to the cgDS_*aaDS*,1_ simulation. Results for the replica simulations are included as dashed lines. Noticeably, the phosphate-binding loop (highlighted in red, denoted as PBL) shows differences in all comparisons, but the differences are decreasing in severity for ClCry4 following the path back to the oxidized form of the FAD cofactor. The final comparison to the cgDS_*cgRP*2,100_ simulation shows only a small difference, which could be considered as a closing of the phosphate-binding loop which had opened in the earlier study.^7^ Additionally, a substantial motion can be observed around residues 440–460 (highlighted in blue, denoted as EEE), which contains a linear EEE motif. The C-terminal region (highlighted in green, denoted as CT) becomes more flexible in the later simulations. Finally, a smaller peak that is also reverting to the DS configuration can be seen around amino acid residues 80–90 (colored in yellow, denoted as OPP). This region is located roughly opposite of W_D_ on the surface of the ClCry4 protein. The different highlighted regions are visualized in their respective color in the protein model structure.

The similarity of the different coarse-grained simulations to the referenced cgDS_*aaDS*,1_ simulation was assessed based on the symmetrized KLD, as visualized in Figure 5: a higher value of the divergence in the plot indicates a greater difference for the respective amino acid residues. Note that the KLD shows a low divergence if both simulations have high residue motility as long as the motion is similar. The plots show four particularly different regions of interest within the ClCry4 structure.

First and foremost, the red highlighted phosphate-binding loop region (PBL) shows a decreased deviation compared to the cgDS_*aaDS*,1_ simulation, as one progresses through cryptochrome’s reoxidation pathway, see Figure 5. Even though, visually, an opening of the phosphate-binding loop was not seen for the cgRPD_*aaDS*,0_ simulation, it is still versatile and differs from the original DS simulation.

A second pronounced region in ClCry4 can be seen in the cgRPD_*aaRPD*,1_ simulations at residues 440–460 (highlighted in blue). The region contains a linear motif of three glutamic acids (EEE) back to back at residues 450–452. In all simulations, except for cgRPD_*aaRPD*,1_, the similarity peak seems neglectable. As the divergence peak arising for residues 440–460 is well separated from the flexible C-terminal (starting at residue 480 onward; green highlights in Figure 5), it is plausible to suggest that in the case of the residues 440–460, one deals with a specific motion characteristic for the RPD state of ClCry4.

The C-terminus of ClCry4, however, seems to undergo an unlocking motion, as it becomes more versatile over the course of the reoxidation cycle. It is rather rigid in the cgDS_*aaDS*,1_ simulation but appears to be more loosely bound over the course of the cgRP_*aaDS*,1_ simulation. It is even most versatile once the simulations reached the fully oxidized form. Notable is that the comparison of the coarse-grained trajectories in Figure 5 shows that once the protein returns to its initial redox configuration, the C-terminal remains flexible, i.e., it shows hysteresis-like behavior.

Several smaller, less specific peaks can also be seen in Figure 5. Notably, these additional peaks are least pronounced in the cgRPD_*aaDS*,0_ and cgDS_*cgRP*2,100_ simulations, which are overall the most similar to the cgDS_*aaDS*,1_ simulation. The most notable of these smaller peaks can be identified around residues 80–90 (highlighted in yellow), which also shows a decrease in difference in the structures during cryptochrome’s back-reaction process. This particular protein region also exhibited some changes upon activation in the all-atom simulation analyzed in the previous study^7^ but was not considered in detail as the difference was minor. In the case of the coarse-grained simulation, the motion is more significant. This specific motion occurs opposite of the surface tryptophans involved in the electron transfer, and, visually, differences manifest as a slight shift of the protein backbone along the surface.

**Figure 6:**
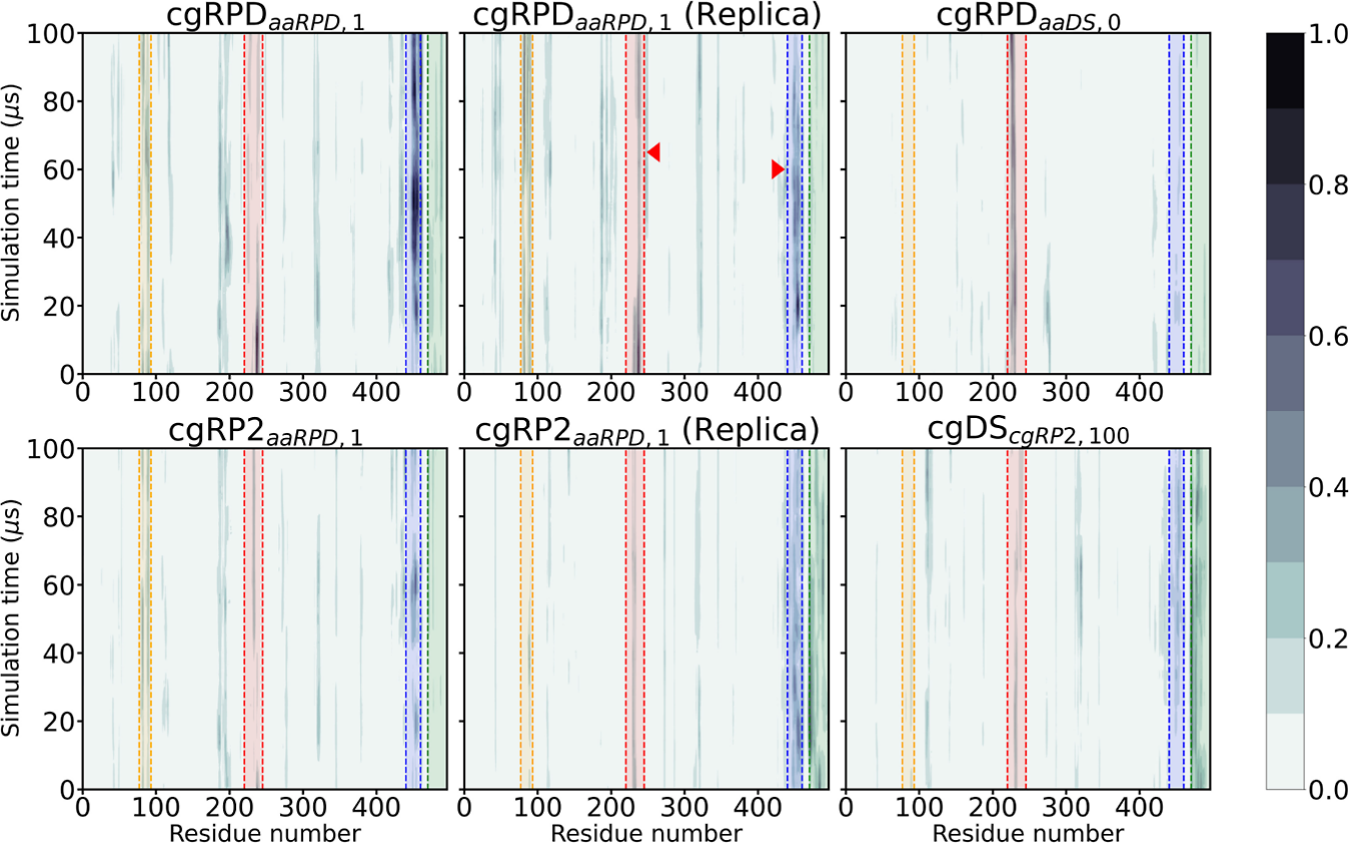
For a time-resolved KLD analysis, the dynamics for each simulation is compared to the evolution of the cgDS_*aaDS*,1_ simulation, as in Figure 5. The plots show the residue number on the abscissa, the simulation time on the ordinate axis. The intensity shows the recorded differences to the cgDS_*aaDS*,1_ simulation for each comparison. The difference has been scaled such that 1.0 corresponds to the largest overall difference observed over all simulations; 0.0 indicates no difference. The four regions highlighted are colored analogously to Figure 5 with the phosphate-binding loop region in red, the region containing the EEE linear motif in blue, the region around residues 80–90 in yellow, and the C-terminal in green. Consistent with the findings of the rmsd analysis, the phosphate-binding loop gate closes in the RPD and RP2 simulations. The EEE region seems to be active once the phosphate-binding loop closed. Interestingly, in the cgRPD_*aaRPD*,1_ replica simulation, it appears as if the phosphate-binding loop acts more versatile once more, once the EEE region shifted back toward the DS configuration. The change is indicated by red arrow tips.

Figure 6 shows the differences in dynamics recorded between all coarse-grained simulations to the cgDS_*aaDS*,1_ simulation in a windowed analysis to study the changes in structural behavior over the simulation time. The protein region containing the phosphate-binding loop is highlighted in red, the region containing the EEE linear motif is highlighted in blue, and the region around residues 80–90 is highlighted in yellow.

Over time, one notes the difference in the phosphate-binding loop mobility decreasing for all simulations, but the cgRPD_*aaDS*,0_ simulation, in which it appears rather persistent after a very swift motion at the beginning. Especially in the cgRPD_*aaRPD*,1_ and cgRP2_*aaRPD*,1_ simulations, it appears as if the protein region around residues 440–460 initiates some motion once the phosphate-binding loop dynamics become similar to its motion present in the closed DS configuration. The putative interaction between the phosphate-binding loop and the 440–460 region is best seen in the cgRPD_*aaRPD*,1_ replica simulation; whenever the difference for the phosphate-binding loop decreased, the motion around residues 440–460 is triggered. Conversely, once the divergence increases again in the 440–460 region, the phosphate-binding loop becomes versatile again, indicated by the red arrow.

**Figure 7:**
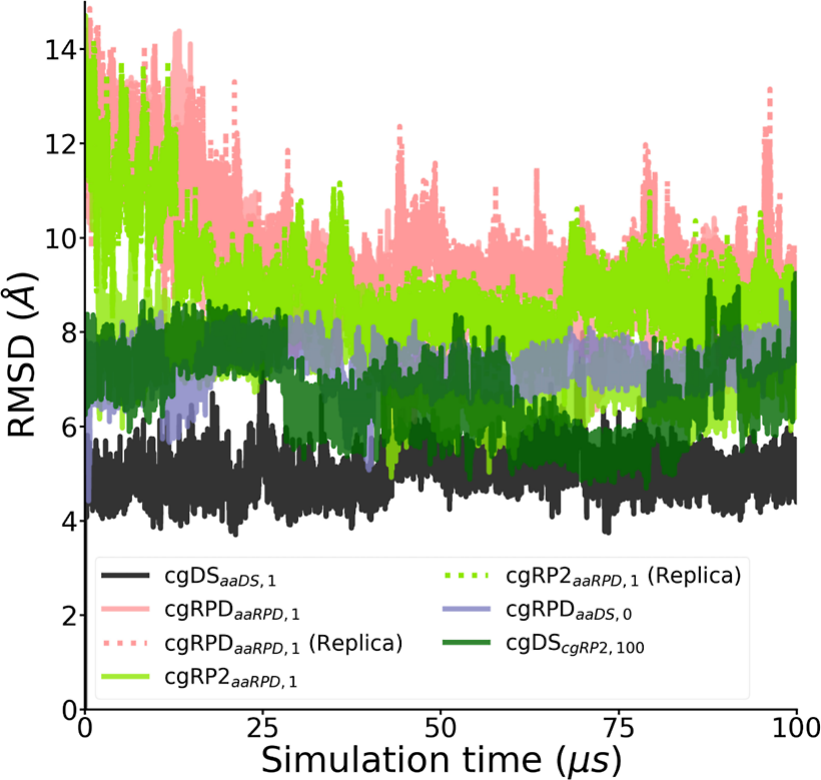
rmsd for the phosphate-binding loop is shown. The loop was found to be versatile in the KLD similarity measure as well as in an earlier all-atom study.^45^ The trajectories were aligned to the full protein structure of cgDS_*aaDS*,1_. Notably, the rmsd decreased straight away for the cgRP2_*aaRPD*,1_ simulation (light green); after 20 μs, it also decreases for the cgRPD_*aaRPD*,1_ (pink). The decrease in rmsd indicates that the phosphate-binding loop moved toward the closed DS configuration.

The pronounced conformational differences in the phosphate-binding loop region in the protein structure warrant a close look at the rmsd of this particular protein region. The rmsd for the phosphate-binding loop indicated differences compared to those in the cgDS_*aaDS*,1_ simulation (Figure 7). Specifically, the phosphate-binding loop in the cgRPD_*aaRPD*,1_ moved back toward its position in ClCry4’s DS conformation after around 20 μs (Figure 7, pink line); the cgRP2_*aaRPD*,1_ simulations underwent a similar rearrangement after 5 or 20 μs (light green line). The reconfiguration of the protein to the cgDS_*cgRP*2,100_ simulation did not introduce further change during the studied time interval. For the cgRPD_*aaDS*,0_ simulation (dark blue in Figure 7), which originated from the closed all-atom DS structure, the phosphate-binding loop did not swing open like a gate, even though it remains versatile. Overall, the rmsd values concur with the analysis obtained from the KLD measure.

**Figure 8:**
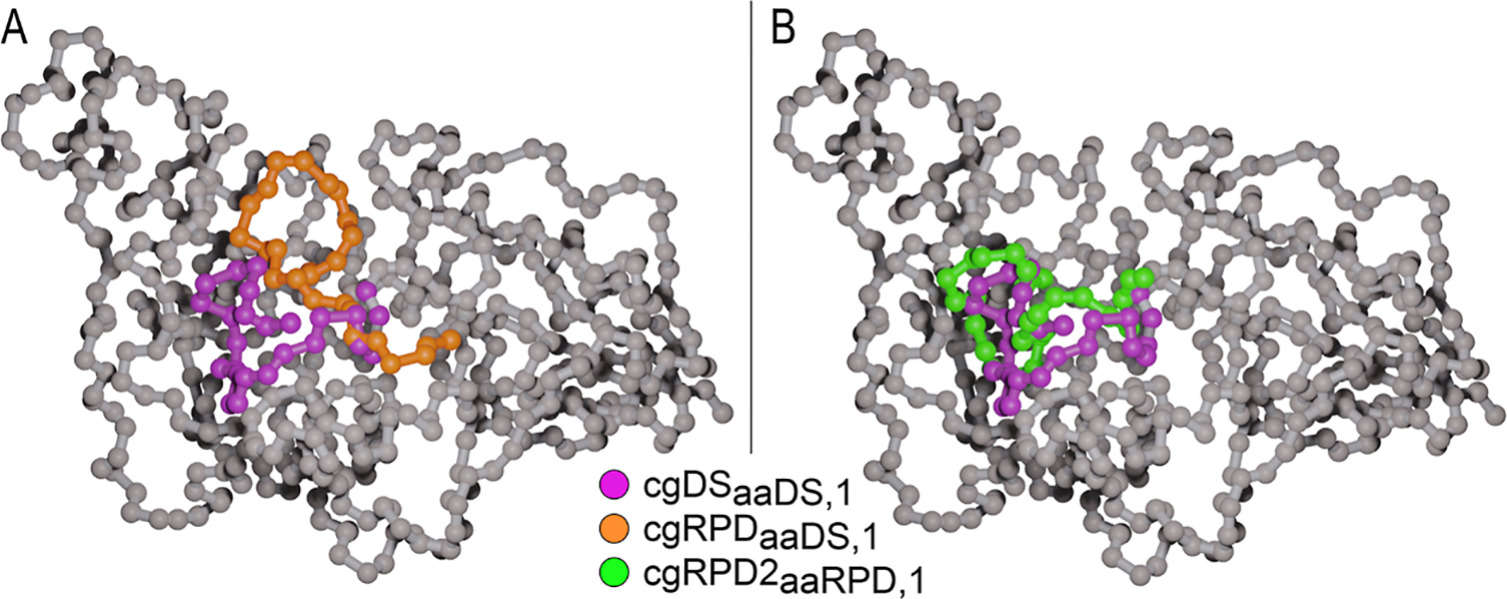
Panels (A,B) both show the backbone beads of ClCry4 (gray). The highlighted region is the phosphate-binding loop displaying the motion it exhibits during different simulations. Panel (A) compares the closed state (purple) to the open state (orange). Panel (B) shows the rapid movement of the phosphate-binding loop toward its closed configuration as exhibited in the cgRP2_*aaRPD*,1_ simulation. The image has been rendered using Blender^46^ with the MolecularNodes Addon.^47^

The overall closing motion of the phosphate-binding loop gate in ClCry4 can be evidently seen through the decreasing of the overall KLD difference computed relative to the closed DS conformation and the corresponding rmsd analysis. In the case of the cgRP2_*aaRPD*,1_ simulation, the closing of the gate happens more swiftly, compared to the cgRPD_*aaRPD*,1_ simulations; the closing process is even more pronounced in the cgDS_*cgRP*2,100_ simulations. The long scale simulations thus support the hypothesis that the phosphate-binding loop gate indeed closes during cryptochrome’s reoxidation process, priming the protein for an additional cycle of photoactivation and signaling. The different conformations of the phosphate-binding loop in different simulations of ClCry4 are visualized in Figure 8. Note that, the phosphate-binding loop gate also closes in the cgRPD_*aaRPD*,1_ simulation, but over a longer time scale than in the cgRP2_*aaRPD*,1_ simulation. In the case of the cgRP2_*aaRPD*,1_ Replica simulation, it takes around 20 μs for the gate to close, but the phosphate-binding loop is overall more similar in terms of the rmsd to the cgDS_*aaDS*,1_ simulation. Here, the faster sampling of a coarse-grained simulation might indicate that a closed and an open conformation are energetically rather similar. It is noteworthy, however, that the opening of the gate in the all-atom simulation happened within a microsecond for one trajectory, while the closing here happened after 20 μs, which is longer than the estimated lifetime of the RPD radical pair.^8^

**Figure 9:**
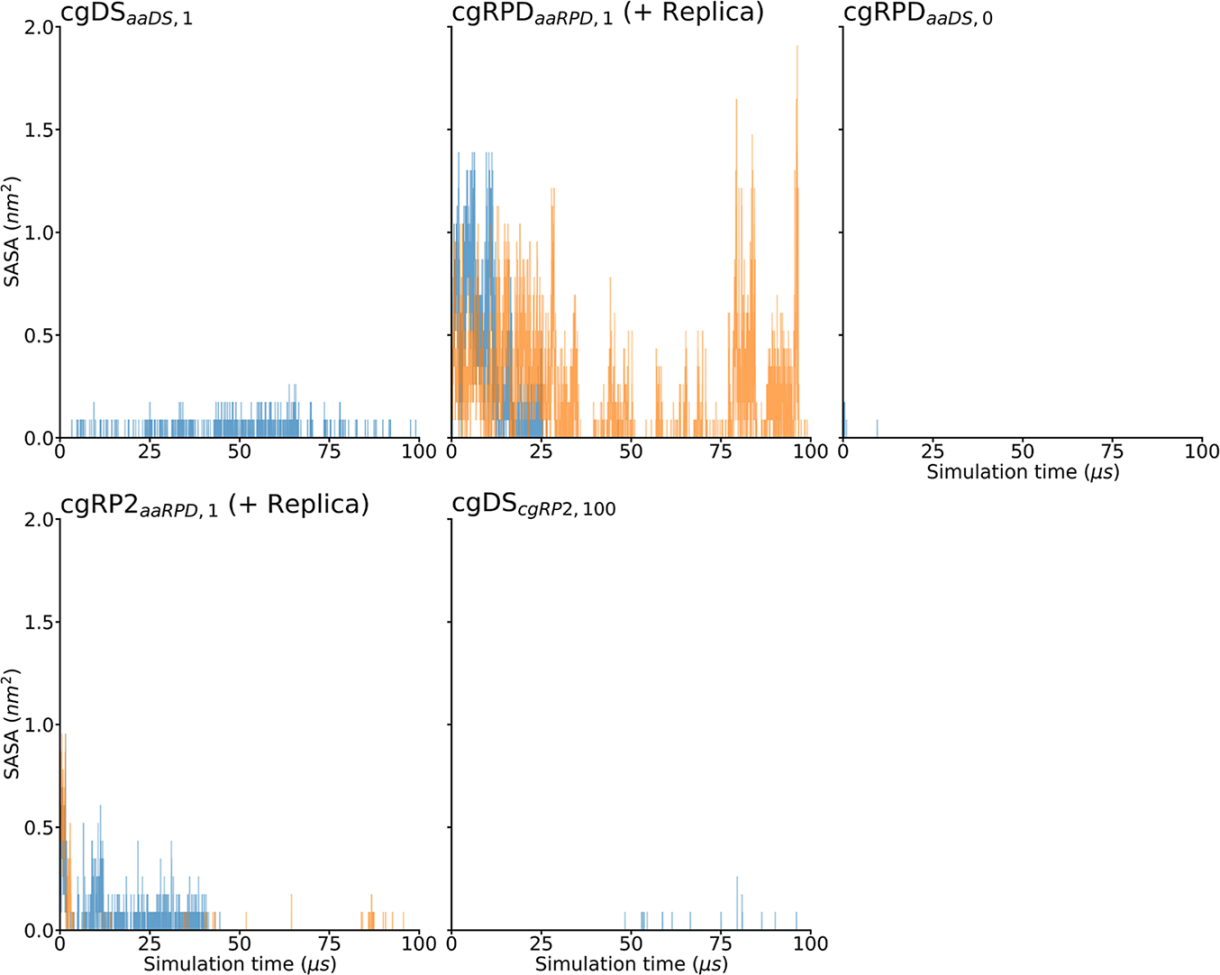
All panels show the SASA for FAD inside ClCry4, computed for the different simulations and analyzed over the 100 μs simulation time (blue). Results for the replica simulations are shown in orange. Notably, the SASA of the FAD decreases over time in the cgRPD_*aaRPD*,1_ and the cgRP2_*aaRPD*,1_ simulations, while it happens much more swiftly in the cgRP2_*aaRPD*,1_ trajectories. The cgDS_*aaDS*,1_, cgRPD_*aaDS*,0_, and cgDS_*cgRP*2,100_ show a constant low SASA value, indicating that no or limited solvent can reach the FAD in these protein conformations.

In order to further characterize the closing motion of the phosphate-binding loop gate, the time evolution of the SASA of the FAD inside the ClCry4 protein is considered, as shown in Figure 9. The analysis of the SASA values shows that the cgDS_*aaDS*,1_, the cgRPD_*aaDS*,0_, and the cgDS_*cgRP*2,100_ simulations do not exhibit an opened phosphate-binding loop, impeding the access of the solvent to the FAD in its binding pocket. On the other hand, a closing of the phosphate-binding loop gate and a reduction in the SASA values can be seen in the cgRPD_*aaRPD*,1_ and the cgRP2_*aaRPD*,1_ simulations, albeit, the gate closes more rapidly in the cgRP2_*aaRPD*,1_ simulations and stays shut. Another observation is noteworthy in the cgRPD_*aaRPD*,1_ replica simulation. After ca. 75 μs of simulation, the SASA values seem to fluctuate again, allowing some solvent to reach the FAD. The change is attributed once more to a motion in the phosphate-binding loop, indicating that the closed and open states are of comparable free energy in the RPD state of ClCry4.

All simulations except cgRPD_*aaDS*,0_ feature a different motion for residues 440–460 compared to the cgDS_*aaDS*,1_ simulation. Based on the analysis of the cgRPD_*aaRPD*,1_ simulation, the motion might be initiated by closing the phosphate-binding loop. This is, of course, a post hoc, ergo propter hoc observation, which, however, might be strengthened by the second opening of the phosphate-binding loop gate in the cgRPD_*aaRPD*,1_ replica simulation, which seems to happen after the region around residues 440–460 shifted back.

**Figure 10:**
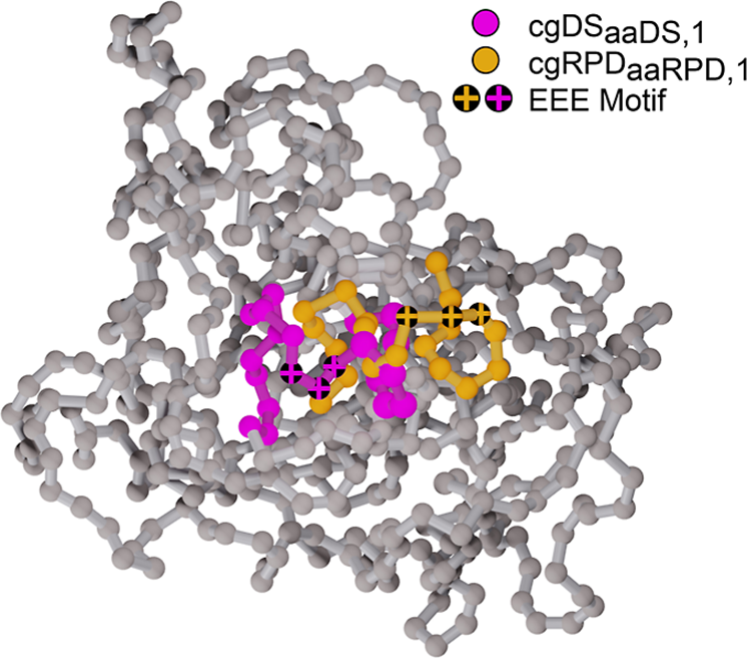
ClCry4 is presented showing the backbone beads (gray) after 80 μs of simulations. The highlighted regions correspond to amino acid residues 440–460 in the cgDS_*aaDS*,1_ (purple) and the cgRPD_*aaRPD*,1_ (orange) simulations. Notably, in the cgRPD_*aaRPD*,1_ simulation, the whole loop shifts to the side displacing the linear EEE motif (crossed residues). Such a structural rearrangement might facilitate the formation of protein complexes for downstream signaling. The image has been rendered using Blender^46^ with the MolecularNodes Addon.^47^

Visual inspection of the cgRPD_*aaRPD*,1_ and cgRP2_*aaRPD*,1_ simulations show that residues 440–460 shift notably to the side of the protein as compared to the cgDS_*aaDS*,1_ simulation, seen in Figure 10. At the far end of the loop, three glutamic acid residues are present back to back, which is a so-called linear motif^48^ and a putative binding sequence which might allow PDZ domain-containing proteins to bind. The name PDZ is derived from the proteins, in which the domain was first discovered (PSD95, DlgA, and Zo-1).^48^ PDZ proteins direct the localization of signaling molecules, arrange the presence of signaling partners,^49^ and are known to anchor proteins in the cytoskeleton of the cell, which might lead to two putative functions. A putative function might be that the rearrangement during the reoxidation process allows the binding or an alteration of the signaling pathway along neighboring proteins. For instance, the PDZ domains can regulate various biological processes, including, e.g., ion channel signaling.^50^ The importance of the EEE linear motif was shown experimentally for Drosophila cryptochrome, in which a version of the protein lacking the motif through truncation of the C-terminal was still able to be light-activated but was unable to respond to a magnetic field. Furthermore, PDZ binding motifs can facilitate the formation of protein complexes. For instance, Bradlaugh *et al.*(49) suggested the formation of protein complexes to transduce the magnetic signal. The conformational changes observed around residues 440–460 might facilitate such a complex formation process.

The importance of the EEE motif motion is further strengthened by the fact that the motif is evolutionarily conserved throughout different bird species (European robin, zebra finch, and chicken) as shown from sequence alignments performed earlier.^44,51^

Lastly, the distances between FAD and the different tryptophans included in the electron transfer cascade were studied. These distances influence the formation and lifetime of the formed spin-correlated radical pair and have previously been studied on the shorter all-atom time scale.

**Figure 11:**
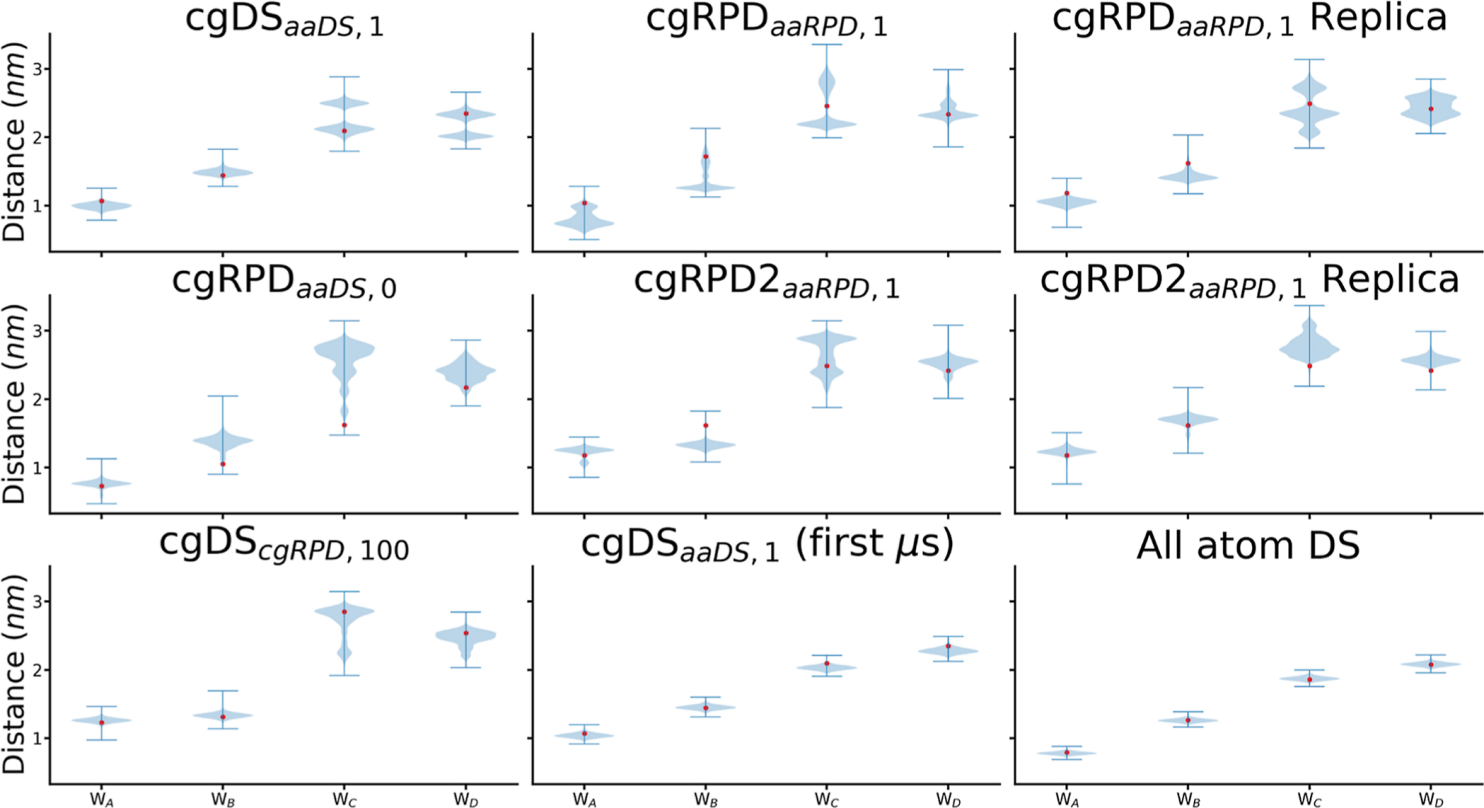
Distributions of the distances calculated between the center of mass of the flavin cofactor and the four tryptophan residue (W_A_, W_B_, W_C_, and W_D_) involved in the electron transfer cascade (see Figure 1A). For each tryptophan, the plot shows the distribution of distances, while the red dot shows the distances for the initial configuration of the protein in the corresponding simulations. The lines extend to the minimum and maximum distance measured for each respective simulation. The plot entitled All atom DS shows the distances calculated by Hanić *et al.*,^44^ while the other plots show the distances for the coarse-grained simulations. Most notably, the distance from FAD to W_C_ and W_D_ are not as clear-cut as in the all-atom case, and even bimodal distributions occur indicating a rearrangement in the side chains of these tryptophan residues. As the all-atom simulation was only conducted for 1 μs, the distances are also shown for the first microsecond of the cgDS_*aaDS*,1_ simulation, showing that the rearrangements indeed only happens on longer time scales.

Figure 11 shows the distances between the FAD and the respective tryptophan residues for different simulations. Additionally, the distances obtained in an earlier study for an all-atom DS simulation are indicated. The all-atom simulation had a total simulation length of 1 μs. To visualize the occurring motion on the all-atom time scale, the first microsecond of the cgDS_*aaDS*,1_ simulation is separately plotted.

Similar to the all-atom MD simulation results, the distances measured between FAD and W_A_ are smaller than those between FAD and W_B_, and they have a similar magnitude as in the all-atom case. Interestingly, the distances between FAD and W_C_ and W_D_ show less clear-cut behavior. The results for the cgDS_*aaDS*,1_ simulation shows a clearly divided bimodal distribution, which is also emerging in the case of the cgRPD_*aaRPD*,1_ simulation. In the case of the coarse-grained simulations, the distances between W_C_/W_D_ and the FAD do not allow for the judgment of which tryptophan residue is closer to the FAD. A visual investigation of the relative orientation of W_C_ and W_D_ residues in the cgDS_*aaDS*,1_ shows a flip in their side chains; one being extended while the other is folded in, after 60 μs. A video of the flipping motion can be found in Supporting Information. Both tryptophans are comparably surface exposed, and the flip results in an approximate interchange of distances to the flavin. Further studies will be required to probe the effects of such a flipping motion on the radical pair mechanism and the magnetic sensitivity of migratory birds.

## Conclusions and Outlook

We performed extensive coarse-grained MD simulations to reach the time scales on which a reoxidation of the FAD cofactor noncovalently bound within ClCry4 might occur. The simulations reveal the closing of the phosphate-binding loop, which therefore closes the activation-reoxidation cycle of the phosphate-binding loop, acting like a gate to the FAD, which is opened upon light-activation and shut again during the reoxidation. Additionally, a distinct protein region that undergoes conformational changes after the closing of the phosphate-binding loop was identified. This region, ranging from amino acid residues 440–460, contains a PDZ domain-binding motif, which could allow the formation of complexes involving ClCry4, anchor the protein in a correct orientation, or facilitate signaling to downstream processes. The exact binding partners might be determined in future studies, while their orientation could be informed through the orientation of the PDZ domain-binding linear motif.

The study also revealed a rearrangement in the two tryptophan residues (W_C_ and W_D_) involved in the formation of the correlated radical pair, in which their side chains switch position while keeping the relevant distances for the pertinent electron transfer processes more or less constant.

Future computational work will have to recreate an all-atom model from the different coarse-grained simulations to validate the structural behavior observed. A special mention should be made of the rearrangements of the tryptophan residue flipping positions and distinguish the feasibility of the protein structure conformation switches. Additionally, the orientation of the linear EEE motif and the shift at residues 80–90 should be considered in the construction of protein clusters and the orientation of potential binding partners of cryptochrome, allowing a signaling process to the birds’ brain. Furthermore, the study calls for an experimental approach to confirm or deny the flip in the tryptophan side chains to judge the possibility in vivo. Secondly, the importance of the EEE linear motif should be assessed for migratory birds.

The coarse-grained approach performed for a single protein in different forms of the FAD cofactor allows long simulations and their analyses involving the reoxidation of the cryptochrome protein. The time scale will allow one to simulate long lasting effects, which might be the key to underpin the magnetoreception phenomenon in migratory birds.

## Supporting Information Available

Illustration of the full activation/deactivation cycle of cryptochrome featuring the different stages and demonstration of the prominent phosphate-binding loop motion observed in the aaRPD, cgRP2_*aaRPD*,1_, and cgDS_*cgRP*2,100_ simulations (MP4)
Illustration of the coarse-grained simulation of ClCry4 featuring the flip in positions of W_C_ and W_D_, four tryptophan residues, TrpA, TrpB, TrpC, and TrpD, corresponding to the residue numbers 395, 372, 318, and 369 shown in red, orange, yellow, and green, respectively, and the flavin cofactor shown in blue (MP4)
